# A 24-Week Physical Activity Intervention Increases Bone Mineral Content without Changes in Bone Markers in Youth with PWS

**DOI:** 10.3390/genes11090984

**Published:** 2020-08-24

**Authors:** Daniela A. Rubin, Kathleen S. Wilson, Camila E. Orsso, Erik R. Gertz, Andrea M. Haqq, Diobel M. Castner, Marilyn Dumont-Driscoll

**Affiliations:** 1 Department of Kinesiology, California State University Fullerton, 800 N. State College Blvd., Fullerton, CA 92831, USA; kswilson@fullerton.edu (K.S.W.); dmendozacastner@gmail.com (D.M.C.); 2 Department of Agricultural, Food, and Nutritional Science, University of Alberta, 8602 112 Street, Edmonton, AB T6G 2E1, Canada; camilaorsso@gmail.com (C.E.O.); Andrea.Haqq@albertahealthservices.ca (A.M.H.); 3 Obesity and Metabolism Unit, Western Human Nutrition Research Center, U.S. Department of Agriculture, 430 W Health Sciences Drive, Davis, CA 95616, USA; erik.gertz@usda.gov; 4 Division of Pediatric Endocrinology, University of Alberta, 8440-112 Street, Edmonton, AB T6G 2B7, Canada; 5 Academic General Pediatrics, University of Florida, Gainesville, 1699 SW 16th Avenue, Gainesville, FL 32608, USA; dumonmd@peds.ufl.edu

**Keywords:** games, parents, home, exercise, bone health

## Abstract

Bone mineral density (BMD) is of concern in Prader-Willi syndrome (PWS). This study compared responses to a physical activity intervention in bone parameters and remodeling markers in youth with PWS (*n* = 45) and youth with non-syndromic obesity (NSO; *n* = 66). Measurements occurred at baseline (PRE) and after 24 weeks (POST) of a home-based active games intervention with strengthening and jumping exercises (intervention group = I) or after a no-intervention period (control group = C). Dual x-ray absorptiometry scans of the hip and lumbar spine (L1-L4) determined BMD and bone mineral content (BMC). Bone markers included fasting bone-specific alkaline phosphatase (BAP) and C-terminal telopeptide of type I collagen (CTx). Both I and C groups increased their hip BMD and BMC (*p* < 0.001). Youth with PWS-I increased their spine BMC from PRE to POST (*p* < 0.001) but not youth with PWS-C (*p* = 1.000). Youth with NSO (I and C) increased their spine BMC between PRE and POST (all *p* < 0.001). Youth with PWS showed lower BAP (108.28 ± 9.19 vs. 139.07 ± 6.41 U/L; *p* = 0.006) and similar CTx (2.07 ± 0.11 vs.1.84 ± 0.14 ng/dL; *p* = 0.193) than those with NSO regardless of time. Likely, the novelty of the intervention exercises for those with PWS contributed to gains in spine BMC beyond growth. Bone remodeling markers were unaltered by the intervention.

## 1. Introduction

Prader-Willi syndrome (PWS) is a complex neurodevelopmental disorder characterized by hypotonia, developmental delay, poor motor competence, and commonly low levels of spontaneous physical activity (PA) [1]. PWS is the best characterized form of syndromic pediatric obesity, and previous studies have shown that adolescents and adults with PWS exhibit lower bone mineral density (BMD) for the whole body and the lumbar spine by dual x-ray absorptiometry (DXA) when compared to sex and body mass index matched controls [2,3]. In adults with PWS, the high incidence of osteopenia and osteoporosis is likely multifactorial and a consequence of the low peak bone mass attained during adolescence and early adulthood [1]. Specifically, hypothalamic hypogonadism, central hypothyroidism, and growth hormone deficiency, common features of the syndrome, may contribute to short stature and poor bone mineralization [1]. This poor bone density was shown in previous studies in which most adults with PWS had not been on growth hormone replacement therapy (GHRT) [2,3].

The reduced levels of PA frequently described in individuals with PWS may also contribute to the low BMD phenotype. In a recent study in youth with PWS who had been on GHRT for at least two years, we showed that youth with PWS showed lower BMD at the hips than height-matched controls with obesity [4]. This finding is potentially due to insufficient ambulatory activity and stimulus for bone formation [5]. Weight-bearing exercise applies mechanical forces on the bones through the ground reaction forces and the contractile activity of the muscles [6]. These physical forces induce strain in the bone and depending on the magnitude of such strain may serve to prevent bone loss or induce bone mass accumulation. This mechanical deformation of the bone is sensed by osteocytes which, in turn, release different molecules such as Wnt, sclerotin, and nitric oxide to regulate the activity of osteoclasts (bone resorption) and osteoblasts (bone formation) [6,7]. The general consensus is that PA is associated with bone accrual and bone strength during childhood but the exact duration and intensity required are yet to be determined [8].

Another approach to monitor bone development other than using body composition techniques is to evaluate changes in blood bone markers related to bone formation and bone resorption. Compared to obese controls, adults with PWS who have not been on GHRT exhibited higher concentrations of bone turnover markers, such as bone-specific alkaline phosphatase (BAP; released by osteoblasts) [2,3] and cross-linked C-terminal telopeptide of type I collagen (CTX-1; bone resorption marker) [3]. Specifically, the increase in the levels of both markers suggests an augmented activity of both formation and resorption. While bone markers change with pharmaceutical interventions [9,10], it is unknown whether blood bone markers are sensitive to PA interventions in PWS.

From what is known, it appears that lean mass and not fat mass is the most important factor for bone health in youth with obesity [11]. This factor can be problematic in PWS as the syndrome is characterized by lower lean mass than expected even in those exposed to GHRT [4]. A recent systematic review and meta-analysis demonstrated that youth with obesity have higher bone mineral content (BMC) and BMD than their normal weight peers [12]. However, from what has been shown, youth with PWS have either lower or similar BMD compared to their obese peers [2,3,4]. This previously mentioned meta-analysis with focus on PA interventions also showed that 50% of the included articles presented with a positive effect on bone health as measured by DXA while bone markers were unaltered [12]. To date, there are no studies that evaluated the effect of a PA intervention on bone parameters in youth with PWS. Thus, this study aim was two-fold: (1) To examine and contrast changes in BMC and BMD in response to a home-based 24-week PA intervention in youth with and without PWS; and (2) to examine changes in blood bone markers (BAP and CTx) in a sub-sample of youth who completed the intervention. It was hypothesized that both groups of youth would demonstrate improvements in BMD and BMC (particularly in the spine, hips, and femoral neck) in response to the intervention. No changes were expected for CTx and possible increases in BAP.

## 2. Materials and Methods

### 2.1. Study Design

#### 2.1.1. Bone Parameters Study Design

Participants were pseudo-randomized to either an intervention group that completed a 24-week PA intervention or a waitlist control group [13]. The control group eventually received the intervention after serving as a control for 24 weeks. The group allocation took place based on the participants’ availability to attend the planned study visits [13]. Measurements took place at baseline and after 24 weeks (post).

#### 2.1.2. Bone Markers Study Design 

This sub-study included measurements completed at pre-intervention (PRE) and post-intervention (POST) for a sub-sample of participants who completed the intervention (regardless of their initial assigned group).

#### 2.1.3. Intervention Description 

At the beginning of the intervention, parents and their children were trained in using a PA curriculum that contained four pre-planned sessions of PA 25–45+ minutes long per week for 24 weeks. Sessions for two days included bone and muscle strengthening exercises and playground-based games. Sessions for the other two days included interactive console-based games using the Nintendo Wii gaming system (one day playing Wii Fit Plus and the other playing Just Dance 2 or 3). The bone and muscle strengthening exercises included resistance exercises using the body weight and jumping. The playground-based games included running and jumping, as well as activities requiring coordination and balance. Participants were provided with all materials and equipment for implementing the curriculum at home. Parents also received communications via phone or email from study staff to check with the progress of the intervention and address any barriers. Every six weeks parents filled in and submitted checklists for the sessions their child completed, which were used to evaluate their child’s adherence with the intervention [14]. The results of the intervention for the PA outcomes and compliance with the PA sessions have been reported elsewhere [15]. In brief, participants’ compliance with the intervention sessions was 68.2% and the intervention did not increase levels of moderate-to-vigorous intensity or total PA [15]. The main intervention goals were to increase levels of PA and improve motor skills. Hence, while it included some high impact and strengthening exercises, it was not designed for bone health.

#### 2.1.4. Participants 

Forty-five children with PWS and sixty-six children with non-syndromic obesity (NSO) participated. The sex distribution of participants included 54% males (PWS, *n* = 25/45, and obese, *n* = 35/66). Youth with NSO presented with a body fat percentage greater than the 95th percentile based on age and sex [16]. PWS diagnosis was confirmed by documentation of appropriate molecular and cytogenetic testing (i.e., chromosomes, FISH 15, DNA methylation, and/or DNA polymorphism studies). The genetic diagnosis for PWS included: deletion (*n* = 20), uniparental disomy (*n* = 8), DNA methylation (*n* = 13), and unknown type (*n* = 4). As GHRT is the standard of care in PWS, 33 youth were currently and had been using GHRT for more than 2 years, nine used GHRT in the past, and three never used GHRT. PWS participants also presented with hip dysplasia (*n* = 2), scoliosis (*n* = 14), and hypothyroidism (*n* = 8). Youth with NSO were excluded if they were currently using lipid-lowering, diabetes, or blood pressure medications or were pregnant.

### 2.2. Ethical Statement

The study protocol was approved by the institutional review boards at California State University, Fullerton HSR-16-0135, University of Florida Gainesville original submission 201702437, and the Human Subjects Research Protection Office from the U.S. Army Research and Materiel Command HRPO A-16501a. All youth signed the approved assent and their parents signed the consent forms. This trial was registered at ClinicalTrials.gov under registration number NCT02058342.

### 2.3. Outcome Variables

#### 2.3.1. Medical History and Anthropometrics

Parents filled out a medical history form that included current or past signs and symptoms of disease and drug and/or supplement use. Pubertal development was estimated from parents’ responses to a modified version of the Pubertal Developmental Scale [17]. Body mass to the nearest 0.01 kg was obtained using a digital scale (ES200L; Ohaus, Pinewood, NJ, USA) with the subject wearing a t-shirt, shorts, and no shoes. Height was measured to the nearest 0.1 cm using a wall-mounted stadiometer (Seca, Ontario, CA, USA) at the end of inhalation. Body mass index (BMI) z-scores were derived from the Centers for Disease Control and Prevention website [18].

#### 2.3.2. Body Composition

All measurements were assessed by DXA with participants positioned following manufacturer’s indication (Lunar Prodigy Advance, GE Healthcare, Madison, WI, USA). Total body fat and lean tissue were expressed as mass in kilograms and as percentage of soft tissue mass. Bone measurements included BMC (grams), and areal BMD (g/cm^2^) for the lumbar spine (L1–L4), left and right hips (hip), bilateral femoral necks and total body minus the head (TBLH). Two technicians completed all scans (lumbar spine, hips, whole body) in the same Lunar Prodigy scanner. The same technician completed all DXA scans for the same participant over time and also completed the subsequent in-software analyses using the Paediatric Encore software version 12.30.008 (GE Healthcare Inc., Madison, WI, USA). The facility least significance change (LSC) in absolute and percent units was calculated using the procedures outlined by the International Society for Clinical Densitometry [19]. The calculated facility LSC for BMD at the L1–L4 were 0.030 g/cm^2^ and 1.713% and for the hip were 0.020 g/cm^2^ and 1.073%.

#### 2.3.3. Nutritional Intake

Parents of youth participants were trained by a registered dietitian with experience in PWS for food record completion. Parents were instructed to log their child’s meals including beverages (e.g., food description and preparation, amount consumed, and consumption location) in real time during one weekend day and two weekdays. Food records were screened to check for compliance in the recording resulting in 63 of 111 food records to be included in the analyses. Food records data and nutritional supplements used were then entered into The Food Processor software, version 10.12.0.0 (ESHA Research, Salem, OR, USA) to determine average daily intake of total kilocalories, vitamin D, and calcium.

#### 2.3.4. Blood Bone Markers

An enzyme-linked immunoassay (ELISA) kit from Quidel Corporation (San Diego, CA, USA) was used to determine BAP concentrations, with an overall coefficient of variation (CV) of 1.63%. An ELISA kit from Immunodiagnostics Systems Inc. (Gaithersburg, MD, USA) was used to determine CTx concentrations with a CV of 2.41%. The LSC was calculated using inter-assay and intra-assay CV with values of 4.52% and 6.67% for BAP and CTx, respectively.

### 2.4. Statistical Analyses

Mean and standard error of the mean values were computed for all participant characteristics, bone and nutrition measurements. Baseline values were compared between treatment groups (intervention vs. control) and youth with PWS and those with NSO using two by two analyses of variance. Generalized estimated equations (GEE) were used to compare changes over time between the control and the intervention groups as well as between those with PWS and those with NSO for all bone parameters (hip, femoral neck, spine, and TBLH BMC, and BMD). Height was included as a covariate for all BMC analyses and baseline values were included as a covariate for all variables [20]. These covariates were selected based on recommendations by the literature and based on differences at baseline between youth or intervention groups in our sample. GEE models were also evaluated to determine changes PRE to POST in the subsample for BAP and CTx. Statistical significance was set at *p* < 0.050. Analyses were conducted using Statistical Package for the Social Sciences version 26.0 for Windows (SPSS, Chicago, IL, USA).

## 3. Results

### 3.1. Participant Baseline Differences

Baseline participant characteristics are presented in Table 1. Youth with PWS were older than those with NSO (*p* = 0.001). Youth in the intervention group were taller than those in the control group (*p* = 0.030). Youth with PWS had lower BMI percentile scores (*p* < 0.001) and z-scores (*p* = 0.002) than youth with NSO at baseline. Youth with PWS were in the prepubertal (*n* = 6), early pubertal (*n* = 12), mid-pubertal (*n* = 17), and late pubertal (*n* = 7) stages. Youth with NSO were in the prepubertal (*n* = 17), early pubertal (*n* = 13), mid-pubertal (*n* = 28), and late pubertal (*n* = 7) stages. There were no differences for body fat % or lean mass between those with and without PWS (*p* > 0.050). Baseline data were analyzed in 111 participants for hip parameters and 107 participants for spine parameters as four participants had metal from spine surgery which precluded spine films. Data were available in 28 youth with PWS and 52 with NSO for TBLH parameters. At baseline, youth with PWS presented lower hip and femoral neck BMC (*p* = 0.030) and BMD (*p* < 0.001) than youth with NSO. Several variables also differed between intervention and control groups including height (*p* = 0.030), hip BMC (*p* = 0.031), femoral neck BMC (*p* = 0.026), femoral neck BMD (*p* = 0.026), spine BMD (*p* = 0.050), and TBLH BMC (*p* = 0.004).

### 3.2. Hip and Femoral Neck Changes

Based on the youth (PWS vs. NSO) or intervention group differences at baseline, all analyses included the baseline values as a covariate. Additionally, height was included as a covariate for BMC outcomes. Because baseline values were included as a covariate, there were no differences between treatments or groups at baseline for any parameter. Table 2 presents outcomes for the hip, femoral neck, spine, and TBLH parameters. There were no group by intervention by time interactions for any of the following parameters: hip BMC (*p* = 0.414), hip BMD (*p* = 0.473), femoral neck BMC (*p* = 0.760) or femoral neck BMD (*p* = 0.258). Youth with PWS showed overall lower hip BMC than those with NSO (21.62 ± 0.13 vs. 22.09 ± 0.08 g, *p* = 0.002). The hip BMC intervention-by-time interaction showed a trend toward statistical significance (*p* = 0.071). Pairwise comparisons showed both the intervention (*p* < 0.001) and control groups (*p* = 0.001) significantly increased from baseline to post in hip BMC (*p* < 0.001); and there were no differences at post (*p* = 0.709). Inspection of means showed a slightly greater increase in the intervention group (7.05% change) than control group (4.75% change). Hip BMD increased from baseline to post regardless of group or intervention (0.873 ± 0.001 vs. 0.894 ± 0.005 g/cm^2^; *p* < 0.001).

Femoral neck BMC increased from baseline to post regardless of group or intervention (3.50 ± 0.000 vs. 3.67 ± 0.022 g, *p* < 0.001). Follow-up pairwise comparisons for the group by time interaction (*p* = 0.021) showed a trend toward statistical significance for an increase in youth with PWS (*p* = 0.064) and a significant increase in the youth with NSO (*p* < 0.001). The means were similar for the groups at post (*p* = 0.151). Femoral neck BMD increased from baseline to post regardless of group or intervention (0.855 ± 0.001 vs. 0.871 ± 0.003 g/cm^2^; *p* < 0.001).

### 3.3. Spine and Total Body Changes

For spine BMC there was a significant group by intervention by time interaction (*p* = 0.050). In youth with PWS, the intervention group showed an increase in BMC from baseline to post (*p* < 0.001) but the control group showed no significant change (*p* = 1.000). For those with PWS, intervention and control groups showed similar spine BMC at post (*p* = 0.266). Youth with NSO showed increased spine BMC between baseline and post regardless of intervention or control (*p* < 0.001 for both). There were no differences between the intervention and control groups at post (*p* = 1.000 for both). There were no significant effects of the intervention, time or group in spine BMD (*p* > 0.109).

For total body BMC, there also was a significant group by time interaction (*p* = 0.032). The pairwise comparisons showed that youth with PWS and those with NSO increased TBLH BMC from baseline to post (PWS: 1320.7 ± 4.7 vs. 1405.9 ± 15.8 g [6.45% change] and NSO: 1314.6 ± 1.5 vs. 1444.5 ± 13.7 g [8.88% change], *p* < 0.001 for both). There were no group differences at post (*p* = 0.358). There was no group by time by intervention interaction (*p* = 0.992) for TBLH BMD. There was a significant group by time interaction for TBLH BMD (*p* = 0.008). Pairwise comparisons showed that youth with PWS increased TBLH BMD from baseline to post (0.872 ± 0.001 vs. 0.885 ± 0.003 g/cm^2^ (1.49% change), *p* < 0.001) and so did the youth with NSO (0.871 ± 0.001 vs. 0.896 ± 0.003 g/cm^2^ (2.87% change), *p* < 0.001). There were no differences at post between youth groups (*p* = 0.097). Table 3 presents all percent changes by group and treatment and the percent improvement of the intervention over the control groups for comparison purposes.

### 3.4. Blood Bone Markers

There were no group-by-time interactions for any bone marker (*p* > 0.425), or time effects (*p* > 0.209). Youth with NSO had overall higher BAP (139.07 ± 6.41 vs. 108.28 ± 9.19 U/L; *p* = 0.006) and similar CTx (2.07 ± 0.11 vs.1.84 ± 0.14 ng/dL; *p* = 0.193) than those with PWS. Values for BAP for youth with PWS were: pre = 107.40 ± 8.73 and post = 109.15 ± 13.43 U/L, and for youth with NSO were: pre = 144.42 ± 7.56 and post = 133.72 ± 7.71 U/L. Values for CTx for youth with PWS were: pre = 1.77 ± 0.13 and post = 1.91 ± 0.19 U/L, and for youth with NSO were: pre = 2.02 ± 0.13 and post = 2.12 ± 0.11 U/L.

### 3.5. Nutritional Intake

Twenty three and 40 food records were analyzed in youth with PWS and with NSO. Youth with PWS consumed lower daily calories (1289 ± 67 vs. 1581 ± 64, *p* = 0.002) and higher calcium (1083.1 ± 133.3 vs. 769.0 ± 30.2 mg, *p* = 0.022) and vitamin D (588.6 ± 143.5 vs. 265.4 ± 33.8 IUD, *p* = 0.028) intakes than those with NSO. Calcium intake increased over time (887.4 ± 70.5 to 964.6 ± 70.0 mg, *p* = 0.017) in both youth groups. Regarding nutritional manipulation, 16/23 and 13/23 youth with PWS reported caloric restriction and using nutritional supplementation (multivitamin and calcium), respectively compared to 3/40 and 7/40 of youth with NSO, respectively.

## 4. Discussion

We hypothesized that completion of a 24-week PA intervention could lead to increases in BMC and BMD in youth with PWS and those with NSO. Our results show that completion of the intervention led to significant increases in BMC in the spine for those with PWS and a small non-significant increase in hip BMC. The intervention had no significant effect in bone parameters in youth with NSO. Percent improvements are discussed below.

In youth with PWS, sustained GHRT not only helps with normalization of height, but also improving BMD [21]. Potentially the role of GH is multifaceted as it may influence bone by normalizing height, increasing lean mass, but also increasing osteocalcin in osteoblasts (bone forming cells) through an IGF-1-mediated pathway. However, as children with PWS fail to go through full puberty, BMD begins to decline despite GHRT, demonstrating the important role for sex hormones inhibiting osteoclast activity [21]. To contribute to the problem is the prescribed hypocaloric diet, which is necessary for weight regulation in PWS [22]. This hypocaloric diet also likely contributes to poor bone mineralization as calcium and vitamin intake are positively related to caloric intake [23,24]. Hence, if individuals with PWS do not supplement their diet with calcium, they likely will not attain the necessary calcium for healthy bone accrual during growth. Thus, all these factors may combine during childhood and adolescence and result in low bone mass and BMD explaining why in this study the group with PWS showed lower BMD and BMC at almost every site compared to youth with obesity.

In youth, recommendations for PA include muscle and bone strengthening exercises at least three days a week in addition to 60 min a day of moderate-to-vigorous PA (MVPA). Reviews evaluating the efficacy of exercise interventions in bone parameters have postulated that effective osteogenic doses can be achieved with interventions that generate a ground reaction force in one leg equal to 3.5 times the body mass delivered three days a week over at least seven months [25]. A recent study by Gabel and collaborators added that the frequency of the stimulus (when of certain intensity such as vigorous intensity [VPA]) and not the total volume is in fact associated with higher bone accrual over time [26]. In prepubertal youth, a meta-analysis including twenty-two studies that used bone loading exercise (resistance training, jumping, and/or high impact activities) showed that the percent improvement for the intervention over the control condition were of 0.8% for total body BMC, 1.5% for femoral neck BMC and 1.7% for spine BMC [27]. However, much larger doses may be needed in youth going through puberty as suggested by the results of this meta-analyses demonstrating no effect of the intervention over the control in the children who were early or post-pubertal [27]. A window of opportunity has been proposed for youth in pre-puberty or early puberty as they experience larger changes compared to when they enter puberty [25]. The hormonal changes that occur during puberty may override the potential effect of exercise on the bone explaining why small changes are observed in interventions in youth during or post puberty [25]. Lastly, the results of a 9-month intervention delivered through the school five days a week showed that children with obesity exhibited less improvement in BMD when compared to those of normal weight [28]. Hence, in the present study, several factors may have contributed to a lack of an effect of the intervention in youth with NSO including an insufficient dose, insufficient intervention length, potentially their pubertal stage as 62% of them were in early or mid-puberty, obesity, and low statistical power (i.e., our study was not a-priori powered to detect changes in bone parameters but powered for other outcomes reported elsewhere [13]). Additionally, compliance with completion of all sessions and activities might have influenced the outcomes [14]. However, the percent improvements, although small, were above the facility computed LSC for BMDs in the hip and spine (1.15% and 2.7%, respectively), indicating that changes in these variables were real and due to the intervention and not measurement errors. Furthermore, these percent changes were above those shown in the previously mentioned meta-analyses [27].

There are not many studies that have evaluated changes in bone parameters in people with PWS. An early study showed no changes in BMD in adults with PWS in response to a four-week exercise intervention delivered at four different times during the year [29]. Likely, the discontinuous nature of the intervention and the lack of routine strengthening or bone building exercises in the routine explain why this early study showed no change in BMD. Thus, our results show promise as we demonstrated increased BMC at the spine by ~8% in the group with PWS in response to this home-based intervention. Our results do not show the same degree of increase in BMC at the hip, but a small non-significant improvement in BMD (~3%) which may be important considering the lower BMD in those with PWS. The risk for low BMD during childhood is problematic because it is such a critical stage for bone acquisition. As BMD during childhood and adolescence tracks over time [30], a poor BMD during growth may increase the risk of future osteoporotic fractures. The small (3.5%) improvement in the hip BMC suggests that the protocol was perhaps insufficient; we only included bone strengthening exercises twice a week. However, the exercises used did not have the dose of jumping interventions (such as jumping from a platform 61 cm high with two feet 100 times) previously tested in youth without PWS [31]. Despite this lack of exercise dose, likely the novelty of the exercises done in addition to a minimum frequency and quantity was sufficient to induce a small effect. Additionally, as we have shown before, youth with PWS were only achieving 30 min of MVPA a day [15] which is less than the recommended 40 min [32].

As more exercise studies are developed for people with PWS, specific exercises must be incorporated to target bone remodeling in those with PWS. The challenge resides in the characteristics needed for the exercise load to have an osteogenic impact [25]. In youth with PWS some limitations exist related to poor muscular strength, running speed, and agility and balance [33]. These factors may limit the ability of individuals with PWS to complete, for example, jumping tasks at a frequency needed. It is possible that to observe larger changes in BMD in the hip, longer interventions with a minimum of three days a week of bone building exercises are needed. In addition, a preparatory period may be allocated to building the minimum motor proficiency to engage in bone-building tasks. We have demonstrated that our intervention strategy improved muscular strength and running speed and agility but did not improve balance in six months [13]. In the action of drop landing or jumping, all three aspects are needed: muscular strength, agility, and dynamic balance. Thus, for youth with PWS to engage in higher impact activities such as running, jumping, or drop landing from elevated platforms [25] basic aspects of their motor skills and proficiency may need to be built first. Of consideration, a plausible approach may be to use a combination of strategies including exercise and whole-body vibration therapy. This combination has been successfully used in youth with severe burns [34].

Our results showed higher BAP in youth without PWS than in those with PWS and no differences in CTx. This lower BAP in those with PWS could be related lower osteoblast activity in those with PWS [35]. The comparable levels of CTx-1 between those with and without PWS is similar to the findings of another study in children and adults with PWS [36]. Our results showed no changes in either BAP or CTx over the 24 weeks. Mostly, changes in bone blood markers have been shown in response to pharmacotherapy [9,10] so the lack of increase in BAP and decrease in CTx is not surprising. The concomitant low concentration of BAP and bone parameters in PWS compared to NSO suggests that perhaps the combination of pharmacotherapy and exercise could be considered to increase bone formation in this population.

By design our group with PWS had an older range of participants compared to those with obesity—this is one reason why we included height as a covariate to assess changes in BMC. Our intervention strategy, while it included bone-building activities twice a week, did not have the recommended optimal dose of three days a week and was not at least seven months long. Our bone blood markers analysis had major limitations as we did not include a control group and the analysis only assesses a snapshot in time. While we tracked changes in diet over time, this was not the main purpose of the study. Therefore, we had no control over dietary changes which resulted in a small increase in calcium intake regardless of youth or treatment allocations. This intervention strategy had a main aim to improve motor proficiency and levels of MVPA and was effective at improving motor proficiency [13] but did not increase ambulation of moderate-to-vigorous intensity [15]. Hence, while some activities might have been novel, likely the osteogenic stimulus might not have been sufficient. Lastly, our study was not powered to detect statistical differences between treatment or youth groups for bone parameters, and we had uneven and relatively small sample sizes for those with PWS. This last aspect, coupled with hip measurements not being the most reliable site for measurement in growing children [37], might have influenced our hip results.

## 5. Conclusions

This study confirmed results from earlier studies showing lower BMC and BMD in youth with PWS compared to those with obesity. It extended previous characterization studies by showing that youth with PWS (most of them on GHRT) showed lower levels of a marker for bone absorption. Importantly, the results of this study showed increased BMC in youth with PWS after completing a novel 24-week home-based intervention that had a twice-a-week muscle and bone building exercise routine. Future studies aiming to improve bone health in PWS should consider longer intervention strategies that first built upon muscular strength, agility, and dynamic balance that will allow for completion of targeted exercises of higher impact for the bone.

## Figures and Tables

**Table 1:** Baseline characteristics of participants with Prader-Willi syndrome (PWS) and with non-syndromic obesity (NSO).

	Intervention		Control		*p*-Value		
	PWS (*n* = 34)	NSO (*n* = 43)	PWS (*n* = 11)	NSO (*n* = 23)	G	I	G × I
Age (years)	10.9 (2.5)	10.0 (1.0)	10.8 (2.3)	9.2 (1.0)	0.001	0.242	0.354
Weight (kg)	58.46 (21.48)	62.98 (19.04)	62.44 (33.18)	59.90 (15.93)	0.828	0.921	0.438
Height (cm)	145.85 (11.84)	148.17 (8.63)	140.99 (13.58)	143.20 (9.11)	0.313	0.030	0.982
BMI z-score	1.719 (0.864)	2.087 (0.468)	1.759 (1.091)	2.322 (0.306)	0.002	0.344	0.502
Body Fat (%)	46.3 (9.9)	44.1 (6.2)	47.0 (9.1)	45.5 (4.8)	0.274	0.528	0.831
Lean Mass (kg)	28.68 (8.51)	32.91 (7.91)	29.82 (12.82)	31.12 (7.29)	0.138	0.861	0.429
Hip							
BMC (g)	19.82 (7.12)	24.07 (6.67)	17.61 (7.296)	20.03 (5.18)	0.022	0.031	0.525
BMD (g/cm^2^)	0.837 (0.151)	0.927 (0.132)	0.778 (0.146)	0.870 (0.116)	0.003	0.053	0.959
Femoral neck							
BMC (g)	3.34 (1.05)	3.89 (0.93)	2.97 (1.20)	3.33 (0.69)	0.030	0.026	0.662
BMD (g/cm^2^)	0.817 (0.149)	0.914 (0.132)	0.742 (0.138)	0.858 (0.108)	0.001	0.026	0.740
Spine							
BMC (g)	36.38 (14.79)	34.89 (9.19)	32.76 (8.31)	29.40 (6.41)	0.309	0.057	0.695
BMD (g/cm^2^)	0.929(0.170)	0.904 (0.138)	0.901 (0.082)	0.812 (0.091)	0.061	0.050	0.297
TBLH							
BMC (g)	1311.7 (580.3)	1438.0 (394.9)	864.2 (214.5)	1185.2 (241.9)	0.064	0.004	0.416
BMD (g/cm^2^)	0.866 (0.129)	0.893 (0.088)	0.788 (0.133)	0.865 (0.068)	0.071	0.066	0.385

Data are presented as mean (standard error of the mean). Abbreviations: BMC = bone mineral content, BMD = bone mineral density, TBLH = total body less head, G = group (PWS vs. NSO), I = intervention treatment allocation (intervention vs. waitlist control), G × I = group by intervention interaction.

**Table 2:** Changes in bilateral hip (*n* = 111), spine (*n* = 107) and total body less head (*n* = 80) bone parameters by intervention groups in youth with Prader-Willi syndrome (PWS) and youth with non-syndromic obesity (NSO).

	Hip		Femoral neck		Spine		TBLH	
	BMC ^1,2,3^	BMD ^2^	BMC ^1,2,4^	BMD ^2^	BMC ^1,2,3,4,5^	BMD	BMC ^2,4^	BMD ^1,2,4^
**Intervention**								
*PWS Pre*	21.22 (0.02)	0.874 (0.001)	3.50 (0.00)	0.855 (0.001)	33.69 (0.13)	0.886 (0.001)	1314.7 (2.9)	0.871 (0.000)
*PWS Post*	22.33 (0.13)	0.906 (0.018)	3.64 (0.03)	0.872 (0.004)	36.38 (0.47)	0.917 (0.006)	1390.9 (18.9)	0.886 (0.004)
*NSO Pre*	21.20 (0.03)	0.871 (0.001)	3.50 (0.00)	0.852 (0.001)	33.54 (0.06)	0.887 (0.000)	1311.3 (2.1)	0.871 (0.000)
*NSO Post*	23.08 (0.18)	0.899 (0.005)	3.73 (0.03)	0.880 (0.006)	36.79 (0.65)	0.917 (0.009)	1443.5 (16.1)	0.897 (0.003)
*Overall Pre*	21.21 (0.02)	0.873 (0.000)	3.50 (0.00)	0.854 (0.000)	33.61 (0.07)	0.887 (0.001)	1313.0 (1.5)	0.871 (0.000)
*Overall Post*	22.70 (0.11)	0.902 (0.009)	3.68 (0.02)	0.876 (0.004)	36.59 (0.39)	0.917 (0.005)	1417.2 (12.6)	0.892 (0.002)
**Control**								
*PWS Pre*	21.27 (0.05)	0.876 (0.002)	3.51 (0.01)	0.857 (0.002)	33.96 (0.24)	0.887 (0.000)	1329.7 (7.6)	0.873 (0.001)
*PWS Post*	21.64 (0.47)	0.883 (0.008)	3.59 (0.08)	0.871 (0.009)	32.71 (1.32)	0.867 (0.043)	1420.9 (25.8)	0.884 (0.006)
*NSO Pre*	21.25 (0.03)	0.873 (0.001)	3.51 (0.005)	0.854 (0.001)	33.77 (0.09)	0.887 (0.001)	1318.0 (3.0)	0.871 (0.000)
*NSO Post*	22.85 (0.27)	0.891 (0.001)	3.71 (0.04)	0.863 (0.007)	36.28 (0.33)	0.919 (0.0007)	1445.5 (22.3)	0.894 (0.005)
*Overall Pre*	21.26 (0.03)	0.874 (0.001)	3.51 (0.01)	0.855 (0.001)	33.86 (0.13)	0.887 (0.001)	1322.4 (5.1)	0.872 (0.001)
*Overall Post*	22.25 (0.27)	0.887 (0.005)	3.65 (0.04)	0.867 (0.006)	34.49 (0.69)	0.893 (0.022)	1433.2 (17.4)	0.889 (0.004)

1 = group effect. 2 = time effect. 3 = intervention × time effect. 4 = group × time effect. 5 = group×intervention×time effect. Abbreviations: BMC = bone mineral content (g), BMD = bone mineral density (g/cm^2^), TBLH = total body less head, Pre = baseline. Estimated means (standard error of the mean) are presented. BMC is adjusted for baseline values and height; BMD is adjusted for baseline values.

**Table 3:** Bone parameters percent change (%) as well as percent improvement (%) of the intervention over the control group presented by youth groups.

	Youth with PWS			Youth with NSO		
	% Change		% Improvement	% Change		% Improvement
	Intervention	Control		Intervention	Control	
*Hip*						
BMC	5.25	1.72	3.53	8.85	7.53	1.32
BMD	3.66	0.80	2.86 *	3.21	2.06	1.15 *
*Femoral neck*						
BMC	3.88	2.22	1.66	6.58	5.70	0.88
BMD	1.99	1.63	0.36	3.29	1.05	2.24
*Spine*						
BMC	7.99	−3.69	11.68	9.70	7.44	2.26
BMD	3.50	3.61	−0.11	3.38	0.68	2.7 *
*TBLH*						
BMC	5.80	6.86	−1.06	10.08	9.67	0.41
BMD	1.72	1.26	0.46	2.99	2.64	0.35

* Percent improvement of the intervention over the control condition is above the facility least significance change (LSC) calculated for hip and spine BMD. Abbreviations: BMC = bone mineral content, BMD = bone mineral density, TBLH = total body less head, PWS = Prader-Willi syndrome, NSO = non-syndromic obesity.
